# Chronic voluntary exercise induces plasticity of noradrenaline-activated dopamine D_1_-like receptor signaling

**DOI:** 10.1186/s13041-025-01219-5

**Published:** 2025-06-16

**Authors:** Katsunori Kobayashi

**Affiliations:** 1 Department of System Neurophysiology, Wakayama Medical University, Wakayama, 641-8509 Japan; 2 Department of Pharmacology, Graduate School of Medicine, Nippon Medical School, Tokyo, 113-8602 Japan

**Keywords:** Exercise, Noradrenaline, Dopamine, Hippocampus, Mossy fiber

## Abstract

Physical exercise has lasting positive influence on mental health. However, its cellular substrate remains to be elucidated. Recently, dopamine D_1_-like receptor activation induced by noradrenaline has been suggested to underlie exercise-dependent augmentation of antidepressant effects. The present study demonstrates that exercise induces a long-term enhancement of this atypical catecholaminergic signaling. Noradrenaline potentiates hippocampal mossy fiber synaptic transmission by activating D_1_-like receptors in mice. Voluntary exercise by wheel running enhanced this noradrenaline-D_1_-like receptor signaling within 5 days. The enhancement of the noradrenaline-D_1_-like receptor signaling did not require the integrity of noradrenergic fibers and was maintained for more than 2 weeks after cessation of wheel running. Notably, the effect of exercise was more robustly seen in D_1_-like receptor signaling activated by noradrenaline as compared with dopamine, indicating particular responsiveness of the noradrenaline-activated D_1_-like receptor signaling to exercise. These results suggest that exercise could exert lasting influence on brain functioning via plasticity of the hippocampal noradrenaline-D_1_-like receptor signaling.

**Supplementary Information:**

The online version contains supplementary material available at 10.1186/s13041-025-01219-5.

Exercise has positive effects on mental health and has been attracting attention as augmentation for antidepressant therapy [1]. While exercise can exert lasting beneficial effects in patients with depression [2], long-term follow-up studies generally showed small effects [3]. Understanding lasting influence of exercise on neuronal functioning in the brain would improve therapeutic treatment for depression and also help to promote mental wellbeing. The hippocampus is one of plausible candidate regions linking exercise with brain functioning [4]. It has recently been shown that noradrenaline activates dopamine D_1_-like receptors (D_1_LRs), most probably D_1_ receptors, at the hippocampal mossy fiber (MF) to CA3 synapse [5–7]. Voluntary exercise and restraint stress synergistically augmented this atypical catecholaminergic signaling, thereby facilitating antidepressant effects in mice [5]. These findings suggest a potential importance of the hippocampal noradrenaline-activated D_1_LR signaling in beneficial effects of exercise on brain functioning. The present study aims to characterize the effects of exercise on the noradrenaline-D_1_LR signaling, with a focus on the persistence of the effects.

Field excitatory postsynaptic potentials (EPSPs) arising from the MF synapse were recorded using mouse hippocampal slice preparations. Noradrenaline potentiates MF EPSPs by activating dopamine D_1_LRs and adrenaline β_1_ receptors [5]. In order to isolate the noradrenaline-D_1_LR signaling, all recordings were made in the presence of the non-specific β receptor antagonist propranolol unless otherwise specified. Several weeks of voluntary exercise by wheel running robustly enhanced synaptic potentiation induced by bath-applied noradrenaline (Fig. 1a and b), confirming the previous result [5]. A significant enhancement was observed at 5 days after starting exercise, and the maximal enhancement was reached in 2 weeks (Fig. 1b). The D_1_LR antagonist SKF83566 completely suppressed noradrenaline-induced synaptic potentiation in mice subjected to 2 weeks of exercise (Fig. 1a), confirming the enhancement of D_1_LR signaling. The effect of exercise on D_1_LR signaling activated by dopamine was also examined. Dopamine potentiates MF synaptic transmission by activating D_1_LRs [6, 7]. Wheel running exercise enhanced dopamine-induced synaptic potentiation as well (Fig. 1c). However, the effect of exercise on dopamine-induced synaptic potentiation was significantly smaller than that on noradrenaline-induced synaptic potentiation (Fig. 1d). These results indicate that the D_1_LR signaling activated by noradrenaline is particularly responsive to exercise.

**Fig. 1:**
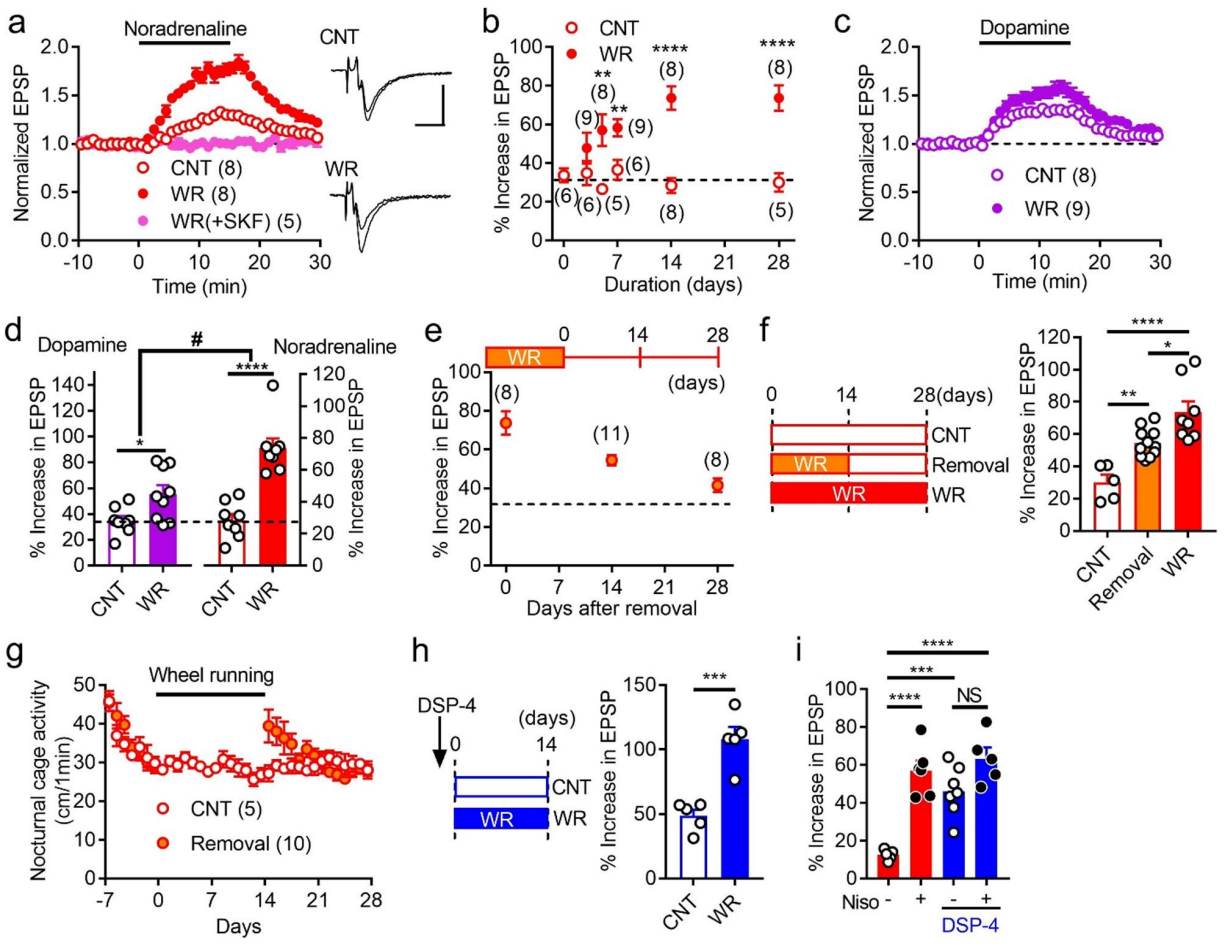
Enhancement of noradrenaline-D_1_-like receptor signaling by exercise. **a** Potentiation of EPSPs at the MF synapse by noradrenaline (10 µM) in control mice (CNT) and after 2 weeks of wheel running (WR). Noradrenaline was applied at the bar in the presence of propranolol (10 µM). In some experiments, SKF83566 (SKF, 200 nM) was supplemented. Sample recordings show averaged field EPSPs before and during noradrenaline application (scale bars: 10 ms, 0.2 mV). **b** Enhancement of noradrenaline-induced synaptic potentiation depends on duration of WR (Day5: Welch’s correction, t_7.572_ = 3.635, **P = 0.0073; Day7: t_13_ = 3.159, **P = 0.0075; Day14: t_14_ = 6.259, ****P < 0.0001). See **f** for statistics of Day28. **c** Effects of 2 weeks of WR on synaptic potentiation induced by dopamine (5 µM). Recordings were made in the absence of propranolol. **d** Enhancement of dopamine-induced synaptic potentiation by WR (t_15_ = 2.612, *P = 0.0196), and a larger effect of WR on noradrenaline-induced synaptic potentiation (two-way ANOVA: substance × WR interaction, F_1,29_ = 5.184, #*P* = 0.0304). Noradrenaline data are same as those of Day14 in **b**. **e** Noradrenaline-induced synaptic potentiation after removal of running wheel. **f** Significant enhancement of noradrenaline-induced synaptic potentiation maintained after cessation of WR. The data of CNT and WR are same as those of Day28 in **b**. One-way ANOVA (F_2, 21_ = 16.68, *P* < 0.0001) followed by Tukey’s test (*P = 0.0141, **P = 0.0072, ****P < 0.0001). **g** A transient increase in nocturnal home cage activity after wheel running exercise. **h** Effects of WR on noradrenaline-induced synaptic potentiation in mice treated with DSP-4 (50 mg/kg) (t_8_ = 5.632, ***P = 0.0005). **i** DSP-4 augments synaptic potentiation induced by a lower concentration (3 µM) of noradrenaline and occludes the effect of the noradrenaline uptake inhibitor nisoxetine (Niso, 1 µM). Mice subjected to 2 weeks of WR were used. Two-way ANOVA (DSP-4 effect: F_1,19_ = 16.35, *P* = 0.0007; Niso effect: F_1,19_ = 38.53, *P* < 0.0001; interaction: F_1,19_ = 7.586, *P* = 0.0126) followed by Tukey’s test (***P = 0.0005, ****P < 0.0001, NS: not significant). All data are presented as means ± SEM with or without individual values. The number of data shown in parentheses represents the number of mice in **g** and slices in other panels

Next, I examined the maintenance of the exercise-induced enhancement of the noradrenaline-D_1_LR signaling. After 2 weeks of voluntary exercise, running wheels were removed. Noradrenaline-induced synaptic potentiation remained significantly elevated at 2 weeks after removal of running wheels and declined toward the control level over another 2 weeks (Fig. 1e and f). Wheel running may have increased basal locomotor activity levels of mice in the home cages, which may support the elevated synaptic potentiation in the absence of running wheels. To test this possibility, home cage activity was monitored before and after the period of wheel running exercise. Indeed, home cage activity increased after exercise, but returned to the control level within a week (Fig. 1g). These results indicate that exercise induces long-term plastic changes in the noradrenaline-D_1_LR signaling.

Exercise increases neuronal activity in the locus coeruleus, a major origin of noradrenergic projection to the hippocampus, and induces noradrenaline release in the hippocampus [8, 9]. Finaly, a role of noradrenergic fibers in the exercise-induced enhancement of the noradrenaline-D_1_LR signaling was examined by using the noradrenergic neurotoxin DSP-4. DSP-4 damages nerve terminals of noradrenergic fibers from the locus coeruleus and causes sustained depletion of noradrenaline in brain regions including the hippocampus [10–12] (see Materials and Methods for details). DSP-4 had no effects on the running activity (Fig. S1). In mice treated with DSP-4, exercise significantly enhanced noradrenaline-induced synaptic potentiation (Fig. 1h). The magnitude of enhancement was comparable to that in mice not treated with DSP-4, indicating that DSP-4 did not affect the exercise-induced enhancement of the noradrenaline-D_1_LR signaling. However, the DPS-4 treatment appeared to increase noradrenaline-induced synaptic potentiation itself in both control and exercise groups (compare Fig. 1h with day14 in Fig. 1b). Since bath-applied noradrenaline is taken up by noradrenergic terminals, actual noradrenaline concentrations in the slices would be lower than those in the bath [5]. The lesion of the noradrenergic fibers by DSP-4 is supposed to impair this uptake activity [12]. Indeed, DSP-4 strongly augmented synaptic potentiation induced by a lower concentration of noradrenaline and occluded the effect of the noradrenaline uptake inhibitor nisoxetine (Fig. 1i). Taken together, these results suggest that the integrity of the noradrenergic projection to the hippocampus is not essential for the exercise-induced enhancement of the noradrenaline-D_1_LR signaling.

The present study has demonstrated that voluntary exercise causes long-term enhancement of the noradrenaline-D_1_LR signaling at the hippocampal MF synapse. Given potential involvement of the noradrenaline-D_1_LR signaling in antidepressive effects [5], the plasticity of this signaling is a plausible candidate for neuronal substrates mediating lasting beneficial effects of exercise on mental health. Exercise activates hippocampal neurons [4]. Since neuronal excitation increases D_1_ receptor expression in the hippocampus [7], exercise may enhance the D_1_LR signaling by increasing D_1_ receptor expression. However, this idea does not explain the differential effect of exercise on noradrenaline- and dopamine-activated D_1_LR signaling. Molecular mechanisms underlying this process remain to be elucidated.

Previous studies have shown that optogenetic stimulation of noradrenergic fibers modulated hippocampal functioning by activating dopamine D_1_LRs [13–15]. These studies suggested that dopamine released from the noradrenergic fibers activated hippocampal D_1_LRs. However, robust activation of hippocampal D_1_LRs by noradrenaline suggests that both noradrenaline and dopamine potentially contribute to D_1_LR activation upon stimulation of noradrenergic fibers [5]. The marked experience-dependent plasticity of the noradrenaline-D_1_LR signaling demonstrated here implies superior importance of noradrenaline over dopamine in hippocampal D_1_LR signaling regulated by noradrenergic fibers.

A limitation of the present study is the uncertain physiological relevance of the micromolar concentrations of noradrenaline used. Noradrenaline concentrations may reach micromolar levels around the noradrenergic terminals, as estimated previously [5]. However, the actual concentrations of noradrenaline in vivo and their spatial dynamics in physiological conditions are unknown. Therefore, while exercise robustly enhanced the noradrenaline-activated D_1_LR signaling in the slice preparations, the result cannot be readily generalized to physiological functions of the noradrenergic system.

## Electronic supplementary material

Below is the link to the electronic supplementary material.

Supplementary Material 1

Supplementary Material 2

## Data Availability

The datasets used and/or analyzed during the current study are available from the corresponding author on reasonable request.
