# Supplementary material for: Chronic voluntary exercise induces plasticity of noradrenaline-activated dopamine D1-like receptor signaling
Source: Mol Brain. 2025 Jun 16;18:51. doi: 10.1186/s13041-025-01219-5 (PMC12172246; doi:10.1186/s13041-025-01219-5)
Supplement: Supplementary file 1 — Supplementary Material 1 [file 13041_2025_1219_MOESM1_ESM.pdf]

Materials and Methods

Animals and housing

Male C57BL/6J mice were purchased from Clea Japan or Charles River Japan. Mice were initially housed in group for 1 or 2 weeks and then transferred to isolated housing at the age of 8 or 9 weeks. Mice were maintained in 14:10 or 12:12 light/dark cycle (lights on at 6:00 A.M. or 8:00 A.M. through 8:00 P.M.). Since differences in light/dark cycles had no clear effects on the results, all data are pooled. Animal use and procedures were in accordance with the National Institute of Health guidelines and approved by the Animal Care and Use Committee of Wakayama Medical University and Nippon Medical School.

Electrophysiological analysis

Mice were decapitated under deep isoflurane anesthesia, and both hippocampi were isolated. Transverse slices (380 μm) were cut from the middle part of the hippocampus using a tissue slicer (7000smz, Campden Instruments Ltd., Leics., UK) in ice-cold saline (see below). Slices were then incubated for 30 min at 30 ˚C and maintained in a humidified interface holding chamber at room temperature before use. Electrophysiological recordings were made in a submersion-type chamber maintained at 27.0 to 27.5 ˚C and superfused at 2 ml/min with recording saline composed of (in mM): NaCl, 125; KCl, 2.5; NaH_2PO_4, 1.0; $NaHCO_3$, 26.2; glucose, 11; $CaCl_2$, 2.5; $MgCl_2$, 1.3 (equilibrated with 95% O_2 / 5% CO_2). Field excitatory postsynaptic potentials (EPSPs) arising from the mossy fiber (MF) synapses were evoked by stimulating the dentate granule cell layer with bipolar tungsten electrodes and recorded from the stratum lucidum of the CA3 region using a glass pipette filled with 2 M NaCl. The amplitude of field EPSPs was measured as described [1]. A criterion used to identify the MF input was more

than 85% block of EPSPs by an agonist of group II metabotropic glutamate receptors, (2S,2'R,3'R)-2-(2',3'-dicarboxycyclopropyl)glycine (DCG-IV, 1 μM). Single electrical stimulation was delivered at a frequency of 0.05 Hz. Noradrenaline was applied in the bath at 10 μM unless otherwise specified. While the physiological concentration range of noradrenaline in the hippocampus is not known, it is estimated to reach micromolar levels around noradrenergic terminals [2]. Since noradrenaline at the low micromolar range does not induce reliable D_1-like receptor-dependent synaptic potentiation in control mice [2], 10-μM noradrenaline was routinely used in the present study. Dopamine was applied at 5 μM. At this concentration, dopamine induces synaptic potentiation that is comparable in magnitude to that by 10-μM noradrenaline. Propranolol, SKF83566 and nisoxetine were added in the bath at least 30 min before application of noradrenaline. All recordings were made using a Multiclamp 700B amplifier (Molecular Devices, Sunnyvale, CA, USA), filtered at 2 kHz and stored in a personal computer via an interface (digitized at 10 kHz). Noradrenaline and dopamine were purchased from Nacalai Tesque (Kyoto, Japan). Propranolol was purchased from FUJIFILM Wako Pure Chemical Industries (Osaka, Japan). DCG-IV, SKF83566 and nisoxetine were purchased from Tocris Bioscience (Bristol, UK).

Home cage activity monitoring

Mice were singly housed in the opaque cage (15 × 25 × 30 cm) equipped with an infrared video camera at the top, and horizontal activity in the cage was continuously monitored. Outputs from the cameras were fed into a personal computer. Images were captured at a rate of one frame per second, and the distance traveled per minute was analyzed online using software based on the public domain ImageJ (ImageJ HC8; O'Hara and Co., Ltd.,

Tokyo, Japan). Since activity of mice during the light period could be affected by external stimuli such as noise made by workers in the animal facility, the activity during the dark period (nocturnal activity) was evaluated in quantitative analyses.

Wheel running

After 1 week of home cage activity monitoring, a plastic running wheel (15 cm in diameter) was placed in the cage. During the period of wheel running, home cage activity was not monitored. Control mice were housed in the same way without running wheels. In the experiments shown in Fig. 1h, Fig. 1i and Fig. S1, mice were housed in standard transparent cages, and a steal running wheel (14 cm in diameter) with a counter (RWC-15, MELQUEST, Toyama, Japan) was used to assess a possible effect of DSP-4 on running behavior.

DSP-4 treatment

DSP-4 was purchased from Cayman Chemical (Ann Arbor, MI, USA), dissolved in saline just before use, and injected intraperitoneally (50 mg/kg) 3 to 4 days before starting wheel running. DSP-4 primarily lesions noradrenergic fibers from the locus coeruleus [3,4], induces a rapid and persistent decrease in the central noradrenaline content [5], and irreversibly inhibits the noradrenalin uptake in brain tissues [3]. DSP-4 does not seem to cause a loss of noradrenergic neurons, but damages their fibers or terminals [6,7]. In the hippocampus, DSP-4 causes nearly complete loss of dopamine-β-hydroxylase immunostaining [4] and sustained depletion of noradrenaline by 80% or more [3,7-9]. A single injection of DSP-4 at 50 mg/kg is commonly used and has been shown to be effective in mice [9].

Statistics

All data are presented as means ± SEM. Statistical analyses were performed using GraphPad Prism version 7.01. Experiments with two groups were compared with unpaired two-tailed Student's t test unless otherwise specified. Experiments with more than two groups were subjected to one-way or two-way ANOVA, followed by the Tukey's test. Statistical significance was set at $P < 0.05$.

References

1. K. Kobayashi, H. Suzuki, Dopamine selectively potentiates hippocampal mossy fiber to CA3 synaptic transmission. Neuropharmacology. 52, 552-561 (2007).

2. Kobayashi K, Shikano K, Kuroiwa M, Horikawa M, Ito W, Nishi A, et al. Noradrenaline activation of hippocampal dopamine D_1 receptors promotes antidepressant effects. Proc Natl Acad Sci U S A. 2022;119:e2117903119.

3. Ross SB, Stenfors C. DSP4, a selective neurotoxin for the locus coeruleus noradrenergic system. A review of its mode of action. Neurotox Res. 2015;27,15-30.

4. Fritschy JM, Grzanna R. Immunohistochemical analysis of the neurotoxic effects of DSP-4 identifies two populations of noradrenergic axon terminals. Neuroscience. 1989;30,181-97.

5. Jaim-Etcheverry G, Zieher LM. DSP-4: a novel compound with neurotoxic effects on noradrenergic neurons of adult and developing rats. Brain Res. 1980;188:513-23.

6 Szot P, Miguelez C, White SS, Franklin A, Sikkema C, Wilkinson CW, et al. A comprehensive analysis of the effect of DSP4 on the locus coeruleus noradrenergic system in the rat. Neuroscience. 2010;166:279-91.

7. Booze RM, Hall JA, Cress NM, Miller GD, Davis JN. DSP-4 treatment produces abnormal tyrosine hydroxylase immunoreactive fibers in rat hippocampus. Exp Neurol. 1988;101,75-86.

8. Harro J, Pähkla R, Modiri A-R, Harro M, Kask A, Oreland L. Dose-dependent effects of noradrenergic denervation by DSP-4 treatment on forced swimming and beta-adrenoceptor binding in the rat. J Neural Transm (Vienna). 1999;106,619-29.

9. Song S, Jiang L, Oyarzabal EA, Wilson B, Li Z, Shih YI, et al. Loss of brain norepinephrine elicits neuroinflammation-mediated oxidative injury and selective caudo-rostral neurodegeneration. Mol Neurobiol. 2019;56:2653-69.
