# Supplementary material for: Chronic voluntary exercise induces plasticity of noradrenaline-activated dopamine D1-like receptor signaling
Source: Mol Brain. 2025 Jun 16;18:51. doi: 10.1186/s13041-025-01219-5 (PMC12172246; doi:10.1186/s13041-025-01219-5)
Supplement: Supplementary file 2 — Supplementary Material 2 [file 13041_2025_1219_MOESM2_ESM.pdf]

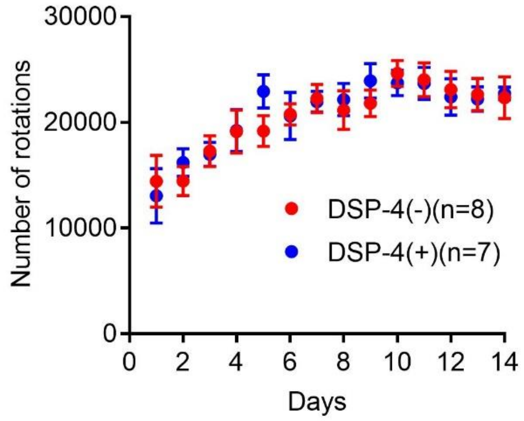

Fig. S1 Effect of DSP-4 on wheel running activity. The number of rotations per day in mice with (+) and without (-) DSP-4 injection is shown. The number of data represents the number of mice.
